# Characteristics of an emerging canine respiratory coronavirus in China

**DOI:** 10.1080/01652176.2025.2574506

**Published:** 2025-10-17

**Authors:** Yunxin Ren, Jian Huang, Xi Chen, Cheng Tang, Hua Yue

**Affiliations:** College of Animal and Veterinary Sciences, Southwest Minzu University, Chengdu, China

**Keywords:** Canine respiratory coronavirus, genetic evolution, phylogenetic analysis, pathogenicity, genome characterization

## Abstract

Canine respiratory coronavirus (CRCoV) is a prevalent pathogen implicated in canine infectious respiratory disease, yet information on its genomic characteristics and pathogenicity remains scarce. To address this situation, we investigated the genetic evolution and pathogenic potential of CRCoV strains circulating in China. Five complete CRCoV genomes (GenBank: PQ725948–PQ725952) were obtained from clinical samples, and phylogenetic analysis showed these strains formed a distinct genetic branch. The evolutionary trees for ORF1ab, HE, and S genes closely mirrored the full genome tree, indicating key roles for these genes in CRCoV evolution. Multiple unique amino acid mutations were identified in the ORF1ab, HE, S, M, and N proteins. Notably, molecular docking analysis suggests that mutations S158F and L161F in the HE lectin domain are associated with improved docking scores, indicating a potential increase in receptor-binding affinity. Consecutive nucleotide deletions in two non-coding regions between non-structural protein genes—which were also identified in strains of a Thai lineage (OQ621707.1–OQ621727.1)—were observed. A CRCoV strain (10^6^ TCID_50_/mL) was isolated, and experimental infection confirmed its ability to induce pneumonia and tracheal cilia loss in dogs. These findings reveal the emergence and unique genetic diversity of a novel CRCoV variant in China, highlighting the need for ongoing epidemiological surveillance.

## 1. Introduction

Canine respiratory coronavirus (CRCoV) is a recognized etiological agent of canine infectious respiratory disease (CIRD) (Day et al. 2020). Common symptoms of CRCoV infection include coughing, sneezing, and the appearance of ocular or nasal discharge and infected dogs are highly prone to secondary bacterial or viral infections that can exacerbate these respiratory symptoms (Mitchell et al. 2013). First detected in kennel-housed dogs in the United Kingdom in 2003 (Erles et al. 2003). Since then, CRCoV infections have been reported in several countries, including the United States (De Luca et al. 2024), United Kingdom (Erles et al. 2003), Italy (Lorusso et al. 2009), Sweden (Wille et al. 2020), China (Lu et al. 2016), Japan (Yachi and Mochizuki 2006), South Korea (An et al. 2010; Lu et al. 2017), Thailand (Poonsin et al. 2023), and New Zealand (More et al. 2021). CRCoV has increasingly been recognized as a globally distributed pathogen. Epidemiologic data show CRCoV detection rates of 0%-99% in global CIRD samples (Erles et al. 2003; Yachi and Mochizuki 2006; Lorusso et al. 2009; An et al. 2010; Lu et al. 2016, 2017; Day et al. 2020; Wille et al. 2020; More et al. 2021; Poonsin et al. 2023; De Luca et al. 2024). This widespread distribution and wide variation in detection rates highlights the global significance of CRCoV in the epidemiology of CIRD.

CRCoV is an enveloped, positive-sense, single-stranded RNA virus classified within the order *Nidovirales*, family *Coronaviridae,* genus *Betacoronavirus*, and species *Betacoronavirus 1* (Erles et al. 2003). Its genome, approximately 30 kb in length, encodes several proteins, including ORF1ab polyprotein, hemagglutinin-esterase (HE), spike (S), envelope (E), membrane (M), and nucleocapsid (N), all of which play critical roles in viral replication and assembly (An et al. 2010; Lu et al. 2017). Genetic and evolutionary analyses have revealed that CRCoV shares close genetic similarities to Bovine Coronavirus (BCoV) and Human Coronavirus OC43 (HCoV-OC43) (Erles et al. 2007). The complete genome nucleotide (nt) similarity of CRCoV with BCoV and HCoV-OC43 strains ranges from 96% to 98% (Wille et al. 2020). In addition, studies have shown cross-reactivity of BCoV with antibodies targeting CRCoV antigens, and the infectivity of BCoV in puppies (Kaneshima et al. 2007; Soma et al. 2008; More et al. 2020). These findings suggest that CRCoV and HCoV-OC43 may represent host-adapted variants of BCoV (Erles et al. 2003; Vijgen et al. 2005).

The evolution of positive-sense RNA viruses, resulting in the emergence of diverse viral strains, serotypes, and subtypes, is primarily driven by mechanisms such as point mutations in coding sequences, small insertions and deletions in both coding and non-coding regions, and homologous RNA recombination (Decaro and Buonavoglia 2008). Consequently, studies based on complete genomes are essential for understanding viral evolution. Currently, 29 complete CRCoV genomes are available in GenBank, including 21 from Thailand, 5 from the United States, and one each from Sweden, South Korea, and China. Previous studies have shown that the Korean CRCoV K37 strain has been identified as a recombinant of the Chinese CRCoV BJ232 and BCoV Quebec strains (Lu et al. 2017); the Thai CRCoV PP158 THA 2015 strain has been reported as a potential parental strain of the CRCoV USA1 strain. Notably, the Thai strains (OQ621707.1-OQ621727.1) exhibit a unique nt deletion at the 4.9 kDa in the non-structural protein gene (Poonsin et al. 2023). However, the availability of complete genome sequence information for CRCoV in China remains limited. Therefore, further sequencing and comprehensive analysis of the complete CRCoV genome are urgently required to enhance our understanding of its genetic evolution.

Despite the CRCoV as a significant respiratory pathogen, research on its pathogenicity in dogs remains limited. To date, the sole relevant study was conducted a decade ago, reported that five CRCoV isolates from geographically distinct regions of the United States and the United Kingdom induced varying degrees of respiratory clinical symptoms, including dyspnea, sneezing, coughing, and nasal or ocular discharge (Mitchell et al. 2013). In 2017, the genome of the CRCoV BJ strain was sequenced from canine respiratory samples in China, although the strain could not be successfully isolated (Lu et al. 2017). Epidemiological surveys in recent years have revealed a high prevalence of CRCoV among dogs in China (6.5%) (Lu et al. 2016). Despite these findings, studies on the genomic characteristics and biological properties of CRCoV strains in China remain scarce. This significantly hinders our comprehensive understanding of the genetic evolution and pathogenic mechanisms of CRCoV strains. Therefore, this study aims to conduct a genetic evolutionary analysis of the CRCoV genome and investigate the pathogenicity of the isolated strains, laying the groundwork for a better understanding of the genetic evolution, epidemiological characteristics, and pathogenic mechanisms of CRCoV in China.

## 2. Materials and methods

### 2.1. Sample collection and CRCoV detection

Between February and April 2024, nasal swab samples were collected from 15 dogs (aged 1–3 months) exhibiting clinical signs of respiratory disease, including persistent coughing and nasal discharge, at the Affiliated Veterinary Teaching Hospital of Southwest Minzu University. The data for each sample are shown in Supplementary Table S1. The nasal swabs were placed into tubes containing 2 mL of pre-chilled Dulbecco’s Modified Eagle Medium (DMEM) supplemented with 1% penicillin-streptomycin and chilled on ice. The nasal swabs were then vortexed in the transport media, and fluid was extracted by pressing the swab against the tube wall. The samples were then centrifuged at 1,0000 rpm for 10 min at 4 °C; the supernatants were aliquoted and stored at −80 °C. Total RNA was extracted using the PureLink^™^ RNA Mini Kit (Invitrogen, Carlsbad, CA, USA), and reverse transcription was performed with random hexamers using the Superscript III RT reverse transcriptase kit (Invitrogen, Carlsbad, CA, USA). The PCR procedure was conducted according to previously reported methods for the detection of CRCoV (Thieulent et al. 2023).

### 2.2. CRCoV complete genome amplification

Next-generation sequencing (NGS) was performed on swab samples that tested positive for CRCoV and met high-quality RNA criteria (RNA concentration ≥20 ng/μL, A260/A280 = 1.8–2.0, and RNA integrity number ≥ 7.0). Only 5 out of the 15 collected samples met all of these criteria, including positivity for CRCoV and sufficient RNA quality for NGS. Library preparation and sequencing were conducted by Tsingke (China). Nucleic acid was fragmented to an average fragment size of 300–500 base pairs (bp), and Paired-end (PE) sequencing (100 bp read) was performed using the Illumina Novaseq 6000 PE150 platform. High-quality sequencing reads were assembled into contigs using IDBA_UD software (v1.1.2), which utilizes the De Bruijn Graph algorithm (Peng et al. 2012). The resulting contigs were analyzed with BLAST (v2.10.0+) against the NCBI non-redundant nucleotide (nt) and viral RefSeq databases to evaluate assembly accuracy and completeness (Camacho et al. 2009). To confirm the genomic sequences of CRCoV, eleven pairs of PCR primers were designed based on the sequences obtained from NGS (Supplementary Table S2). The amplified PCR products were purified, cloned into the pMD19-T vector (TaKaRa, Dalian, China), and subsequently sequenced commercially at Tsingke (Chengdu, China) using the Sanger method.

### 2.3. Genomic characterization: phylogeny, recombination, and non-coding regulatory elements

Sequence assembly and multiple sequence similarity analyses were conducted using the SeqMan and MegAlign programs of DNASTAR 7.0 software (DNASTAR Inc., Madison, WI, USA). Multiple sequence alignments and phylogenetic analyses were performed with the MEGA X software (v10) to construct a maximum-likelihood (ML) phylogenetic tree. The substitution model for the constructed phylogram was automatically selected by the software using the ‘Find Best DNA/Protein Models’ option. For recombination analysis, we utilized a previously published dataset (Lu et al. 2017; Poonsin et al. 2023). Recombination events in the aligned sequences were identified using the Recombination Detection Program 4.0 (RDP 4.0, version 4.96). Potential recombinant strains were further confirmed through similarity plots and bootscanning analyses with SimPlot v3.5.1 software. Furthermore, to investigate the non-coding regulatory elements within the identified deletion regions of the CRCoV genome, a comparative computational analysis was conducted using RegRNA 3.0 (Chang et al. 2013). This analysis specifically aimed to identify and compare a comprehensive set of regulatory elements, including: Ribosome binding sites (RBS), AU-rich elements (ARE), Untranslated region (UTR) motifs, C-to-U RNA editing sites, Riboswitches, Cis-regulatory elements of ERPIN, Cis-regulatory elements of Rfam, and Functional RNA sequences.

### 2.4. Selection pressure analysis

To investigate the evolutionary pressures acting on CRCoV, we utilized all available sequences from the GenBank database, including 42 S genes and 39 HE genes sequences from 2005 to 2024). We employed a codon-based maximum likelihood method to calculate the ratio of non-synonymous (dN) to synonymous (dS) substitutions per site. A dN/dS ratio exceeding 1 generally indicates positive or diversifying selection, while a ratio below 1 suggests negative or purifying selection. However, it should be noted that in datasets with very short evolutionary distances or limited sample sizes, dN/dS > 1 may be inflated due to transient polymorphisms or random genetic drift (Kryazhimskiy and Plotkin 2008). Nevertheless, to robustly identify signals of positive selection, we mainly relied on site-specific analyses using four complementary methods: Fixed-effects Likelihood (FEL), Mixed-effects Evolutionary Model (MEME), Single-Likelihood Ancestor Counting (SLAC), and Fast Unconstrained Bayesian AppRoximation (FUBAR). These analyses were conducted *via* the online tool Datamonkey (www.datamonkey.org). All methods consistently identified specific loci under positive selection, underscoring their evolutionary significance.

### 2.5. Protein structure prediction, mutational analysis, and molecular docking

To determine protein tertiary structures, a hierarchical approach was employed. For proteins with identifiable homologous templates, homology models were generated using SWISS-MODEL (http://swissmodel.expasy.org/) (Schwede et al. 2003). Conversely, for targets lacking suitable experimental templates, de novo protein 3D structure prediction was performed utilizing the online tool AlphaFold (https://cosmic-cryoem.org/tools/alphafold/). A simulated model of the mutated amino acid (aa) was generated with the Wizard mutagenesis module in PyMOL (The PyMOL Molecular Graphics System, Version 2.0 Schrödinger). To predict the effects of mutations on protein stability and flexibility, analyses were conducted using DynaMut (http://biosig.unimelb.edu.au/dynamut/) (Rodrigues et al. 2018). The free energy change (ΔΔG) between the wild-type and mutant protein structures serves as an indicator of stability, with ΔΔG values greater than zero suggesting stabilization, while negative values indicate a destabilizing effect. Additionally, the difference in vibrational entropy (ΔΔS _Vib_ ENCoM) assesses protein flexibility, with values above zero indicating increased flexibility while negative values signal reduced flexibility. The O-acetylated sialic (PubChem CID: 24801866) was retrieved from the PubChem database (https://pubchem.ncbi.nlm.nih.gov) as a docking ligand (Zeng et al. 2008). Ligand and protein preparation for molecular docking was performed using AutoDock Vina (version 1.1.2). PyMOL software was used to separate the original ligand and protein structure, dehydrate, and remove organic matter. AutodockTools enabled hydrogenation, charge verification, AD4 atom type assignment, and docking grid box construction. After docking with Vina, the scores of protein-ligand docking combinations were calculated. Post-docking analysis included scoring protein-ligand interactions, followed by three-dimensional and two-dimensional force analysis and visualization using PyMOL and Discovery Studio.

### 2.6. Virus isolation and identification

CRCoV-positive samples were selected for virus isolation using Human colon carcinoma cell lines (HCT-8), as previously described (Erles et al. 2007). Supernatants were passaged twice blindly at 7-day intervals post-inoculation until cytopathic effects (CPE) were observed. Cultures exhibiting CPE were harvested and confirmed by quantitative real-time RT-PCR (qRT-PCR). The virus was purified through three rounds of plaque purification, and the viral titer (TCID_50_/mL) was determined in HCT-8 cells using the Reed–Muench method (Reed and Muench 1938; Kisch and Johnson 1963).

For Indirect Immunofluorescence Assay (IIFA), HCT-8 cells were seeded as a low-density monolayer and incubated with viral aliquots at a multiplicity of infection of 0.1 for 1 h at 37 °C to facilitate viral adsorption. The medium was replaced with serum-free DMEM, followed by a 48-hour incubation. Cells were fixed in pre-chilled acetone (−20 °C) for 30 min, washes three times with phosphate-buffered saline (PBS), and blocked with 3% bovine serum albumin in PBS for 1 h to minimize non-specific binding. Cells were then incubated with a primary rabbit anti-BCoV whole-virus polyclonal antibody (1:1000, developed in our lab), for 30 min at 37 °C, followed by a secondary fluorescein isothiocyanate (FITC)-conjugated anti-rabbit IgG antibody (1:2000) for 2 h at 37 °C. Cell nuclei were stained with 4′,6-diamidino-2-phenylindole (1:2000) for 10 min. Fluorescence signals were observed and imaged using a fluorescence microscope.

For transmission electron microscopy (TEM) analysis, CRCoV-infected HCT-8 cells in 60 mm dishes were harvested when CPE reached 80%-90%. Cells were trypsinized, centrifuged at 2,000 rpm for 3 min at 4 °C, and fixed in 2.5% glutaraldehyde. Following dehydration with acetone, cells were permeabilized and paraffin-embedded, and 60 to 90 nm sections were cut. Sections were stained with uranyl acetate for 10 min and lead citrate for 1 min at room temperature, then examined under a TEM.

### 2.7. Experimental infection of dogs

This study aimed to characterize the acute pathogenicity of CRCoV isolates in a canine model during the early infection phase, by conducting artificial infection experiments as previously described (Mitchell et al. 2013). Six clinically healthy, 12-week-old Beagle dogs were purchased from DOSSY Experimental Animals Co. Ltd. (Sichuan, China). All dogs tested negative for Canine distemper virus (CDV), Canine adenovirus type 2 (CAV-2), Canine parainfluenza virus (CPIV), Canine herpesvirus (CHV), Canine influenza virus (CIV), Canine respiratory coronavirus (CRCoV), Canine pneumovirus (CnPnV), *Mycoplasma cynos*, and *Bordetella bronchiseptica* nucleic acid (Chalker et al. 2004; More et al. 2021; Thieulent et al. 2023). The dogs were housed in temperature-controlled isolation rooms under strict biosecurity measures, which included disinfection of all items before entry, and a pasteurized diet to prevent contamination. Three dogs were challenged with CRCoV isolates (infection group), while the remaining three received uninfected cell culture supernatant as controls (control group). Intranasal inoculation was performed on days 0 and 1 following sedation with xylazine hydrochloride, administering 1.0 mL of virus or control solution (0.5 mL per nostril). Subsequently, two groups were housed in strict isolation.

Daily general health observations, including assessments of general appearance, breathing rate, sneezing, coughing, ocular and nasal secretions, were performed for each dog. Nasal and pharyngeal swabs were collected daily from the infection group for qRT-PCR analysis to monitor viral shedding. To specifically characterize the acute pathogenicity profile of CRCoV isolates, the observation period was set at 14 days post-infection (DPI) in alignment with established protocols for preliminary pathogenesis studies of CRCoV (Mitchell et al. 2013). On the 14 DPI, all dogs in both the infected and control groups were subjected to humane euthanasia. Necropsies were performed immediately, and samples from the lung (apical lung lobe), trachea, nasal turbinate, nasal septum, lung lavage, nasal tonsil, heart, liver, spleen, kidneys, duodenum, jejunum, ileum, cecum, colon, and rectum were collected. Samples were frozen at −80 °C for viral load quantification by qRT-PCR. Additionally, representative respiratory-related tissues, including the lung (apical lung lobe), trachea, nasal turbinate, nasal septum, and nasal tonsil, were fixed in 10% neutral buffered formalin for histopathological examination. All procedures were conducted at a contracted research facility in compliance with national animal welfare regulations. The study protocol was approved by the local ethics review board.

## 3. Results

### 3.1. Complete genomic characterization of CRCoV

**Figure 1.**
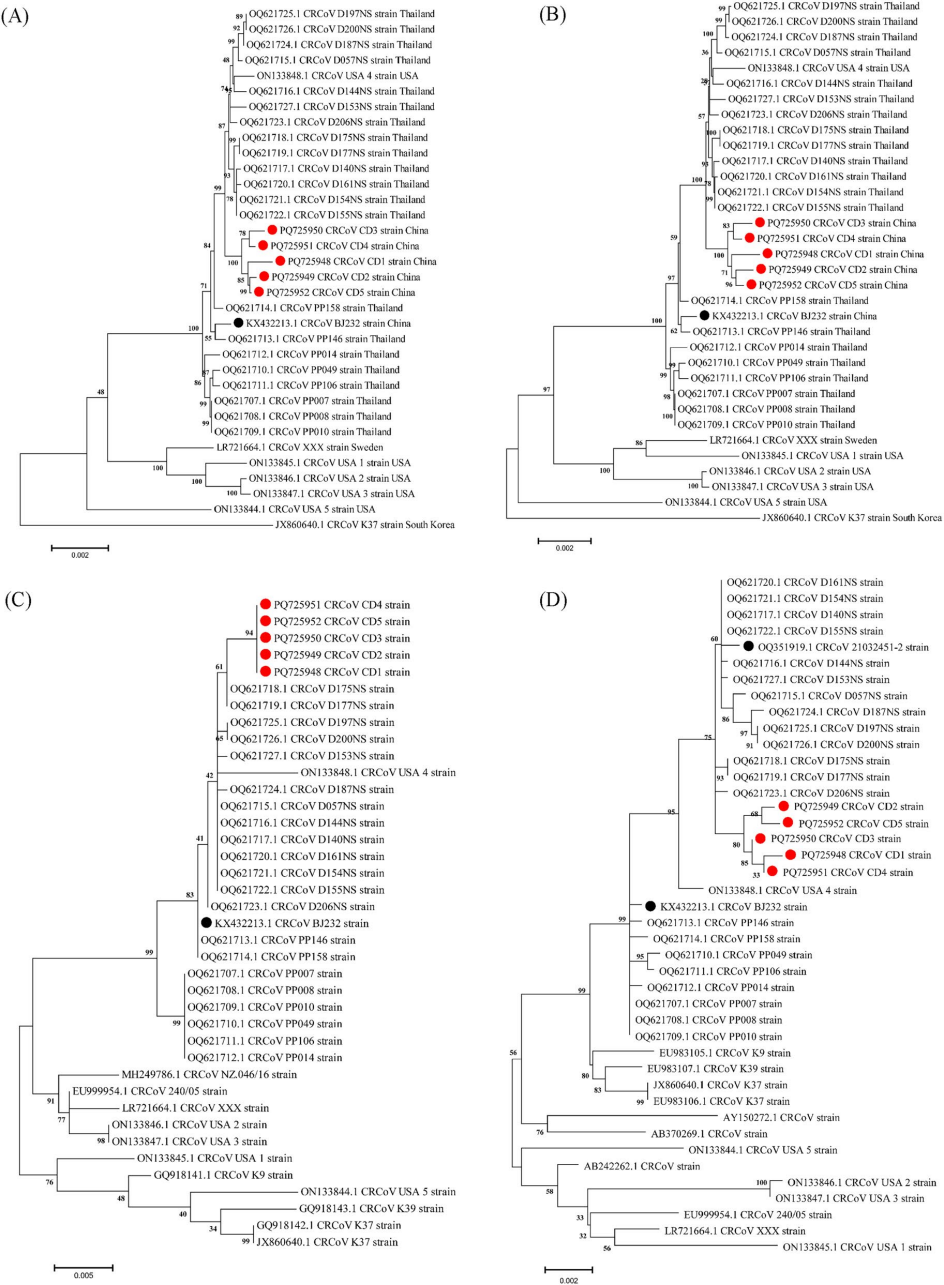
The Phylogenetic trees of CRCoV strains based on all the sequences. (A) Phylogenetic tree of the complete genome. (B) Phylogenetic tree of the ORF1ab gene. (C) Phylogenetic tree of the HE gene. (D) Phylogenetic tree of the S gene. Different colors of symbols indicate the CRCoV strains from China, with the ● symbol representing the CRCoV strain from this study and the ● symbol representing the other Chinese strain. The best-fit substitution model for the phylogram constructed based on the nearly complete coding genome is general time reversible with gamma distribution and invariant sites (GTR + G + I), Tamura-Nei 93 with gamma distribution and invariant sites (TN93 +G + I) for ORF1ab, HE, and S genes. All sequences were analyzed using the maximum likelihood method with 1,000 bootstrap replicates.

Five nearly complete genomes of the CRCoV strains, named as CRCoV CD1, CD2, CD3, CD4, and CD5 (GenBank accession numbers: PQ725948–PQ725952) were obtained from clinical samples and validated by Sanger sequencing. The genome lengths were 30,893 nt, 30,854 nt, 30,852 nt, 30,855 nt, and 30,856 nt, respectively, and all contained complete open reading frames (ORFs). The nt similarity among the five genomes ranged from 99.7% to 99.9%, while the nt similarity to all complete CRCoV genomes in the GenBank database ranged from 97.5% to 99.7%. These genomes shared the highest nt identity (99.5% to 99.7%) with the CRCoV D140NS strain (OQ621717.1), identified in Thailand in 2021. Phylogenetic analysis based on all complete CRCoV genomes, ORF1ab, and S genes revealed that the five strains were mainly clustered into a large branch with Thai CRCoV strains identified between 2021 and 2022 (OQ621715.1– OQ621727.1), which were genetically distant from the Chinese CRCoV strains. However, the five CRCoV strains clustered into a smaller branch (Figure 1A,B,D). Similarly, phylogenetic analysis of the HE gene showed that the five CRCoV strains clustered in a large branch with the Thai CRCoV D175NS and D177NS strains (OQ621718. 1 and OQ621719.1), also genetically distant from the Chinese CRCoV strains, while forming a smaller branch (Figure 1C). In contrast, phylogenetic trees constructed using the E, M, and N genes did not reveal unique evolutionary trends for these strains (Supplementary Figure S1). Recombination analysis showed no evidence of recombination events in the complete genomes of the CRCoV strains analyzed in this study.

**Figure 2.**
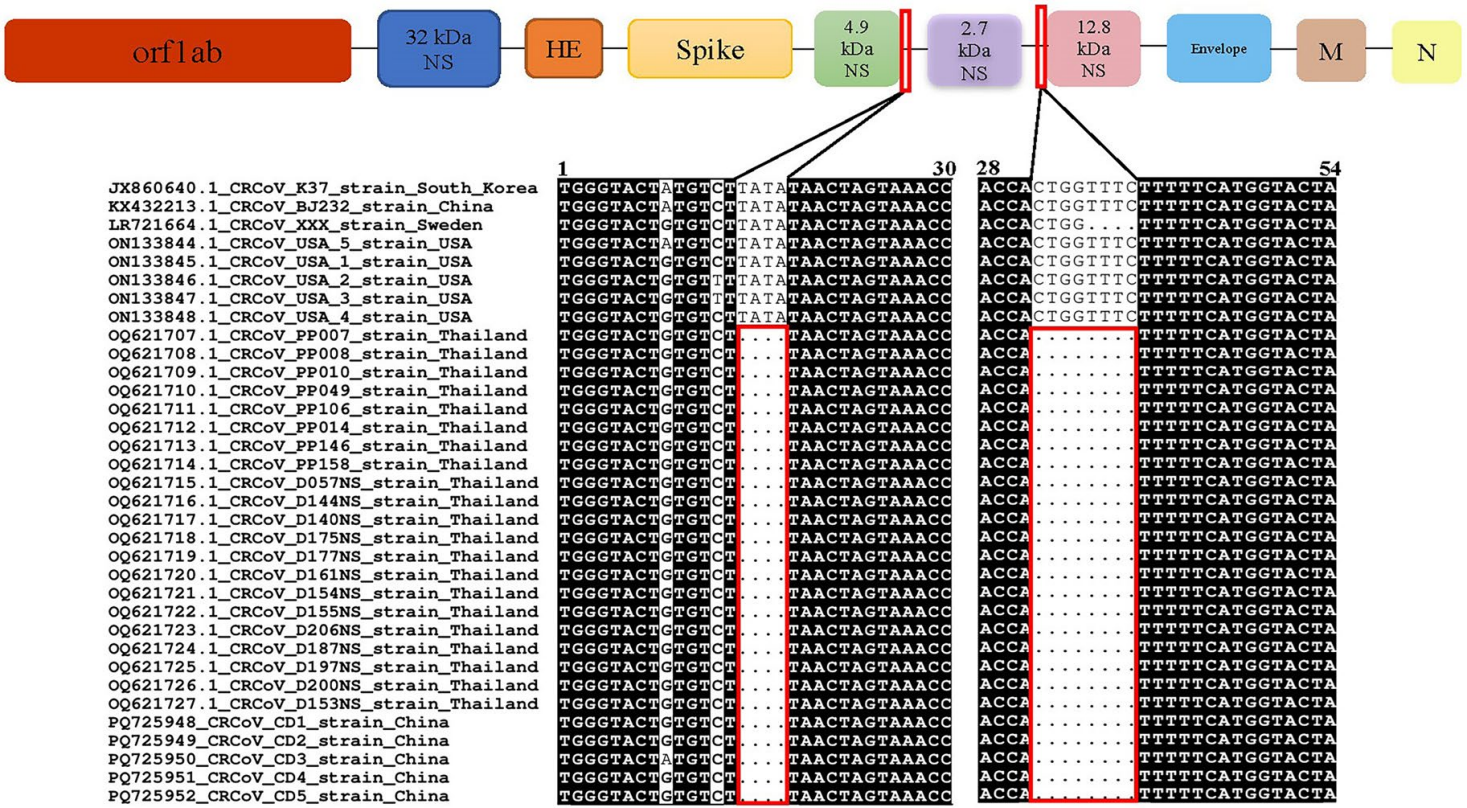
Molecular analysis of CRCoV and reference strain genomes in China. All red boxes represent locations where nt deletions occur.

Further sequence analysis revealed deletions in the non-coding region between the 4.9 and 2.7 kDa regions, as well as another deletion in the non-coding region between the 2.7 and 12.8 kDa regions, with deletion lengths of 4 nt and 8 nt, respectively (Figure 2). These nt changes were unique to the Chinese CRCoV strains analyzed in this study and to Thailand CRCoV strains sequenced from 2021 to 2022 (OQ 621707.1-OQ 621727.1). Notably, the Thai CRCoV strains from 2021 to 2022 exhibited a 10 nt deletion in the 4.9 kDa non-structural region, which was absent in the five CRCoV genomes analyzed in this study.

Analysis of non-coding regulatory elements, focusing on the deletions identified in the CRCoV CD1-CD5 strains, revealed significant alterations. For the deletion within the 4.9 and 2.7 kDa non-coding region, a reduction in the number of predicted Core Promoter Elements (from 5 to 2) was observed post-deletion. This was accompanied by a decrease in their binding stability, as evidenced by an increase in the average minimum free energy (from −4.6 to −4.4). Similarly, the deletion within the 2.7 and 12.8 kDa non-coding region resulted in a slight decrease in the count of Core Promoter Elements (from 30 to 29), alongside a notable reduction in their binding stability (average minimum free energy shifted from −11.573 to −9.924). Detailed data supporting these findings are provided in Supplementary Table S3. In addition, unique aa mutations were identified in the ORF1ab, HE, S, M, E, and N proteins of the CRCoV CD1 to CD5 strains (Table 1). Among these, the ORF1ab protein mutation (K1866R) and the HE protein mutations (P62S, S158F, and L161F) were common to all five strains.

**Table 1.** Analysis of aa mutations in CRCoV CD1 to CD5 strains.

Proteins	CRCoV strains				
	CD1	CD2	CD3	CD4	CD5
ORF1ab	H1766Y, **K1866R**, F2848V, A2903V, Y6809C, K6810Q, M6816V, E6817D, S6820F	P1125S, H1766Y, **K1866R**, D1941N, I6073N	**K1866R**, A2903V, P/S5538A, T5827A, D5829A, G5833S, E5835D	**K1866R**, A2903V, A5992N	H1766Y, **K1866R**
HE	**P62S**, **S158F**, **L161F**	**P62S**, **S158F**, **L161F**	**P62S**, **S158F**, **L161F**	**P62S**, **S158F**, **L161F**	**P62S**, **S158F**, **L161F**
S	I/S510T	–	V132L, I/S510T	V132L, I/S510T	M/T74K, T972I
E	–	–	–	–	–
M	–	I38N, Q41R, F42L, G43V, T45P, S46N, R47C, S48N, F50I	–	–	–
N	–	–	R289I, P308T, A320G, S338C	R107, A112P	G83V, R122L, P158T

*Note:* The single-letter abbreviations for amino acids were used;/represents the different amino acid residues before the mutation; - represents no mutation; Bold font indicates unique aa mutations shared by five CRCoV strains.

### 3.2. Complete ORF1ab, S, and HE gene of CRCoV CD1-CD5 strains

The complete ORF1ab genes of the CRCoV CD1-CD5 strains have a full length of 21,285 bp and encode 7,093 aa. The nt similarity among the ORF1ab genes of the five strains in this study ranges from 99.8% to 99.9%, while the aa similarity ranges from 99.8% to 99.9%. In comparison, the nt similarity with all complete ORF1ab coding sequences in GenBank ranges from 98.5% to 99.9%, and the aa similarity ranges from 98.3% to 99.9%. Furthermore, unique aa mutations were identified in the ORF1ab of the five strains, as detailed in Table 1.

The complete S genes of the CRCoV CD1-CD5 strains have a full length of 4,092 bp and encode 1,363 aa. The nt similarity among the S genes of the five strains in this study ranges from 99.8% to 99.9%, while the aa similarity ranges from 99.5% to 99.6%. Compared to all complete S coding sequences in GenBank, the nt similarity ranges from 98.2% to 99.8%, and the aa similarity ranges from 99.1% to 99.6%. In addition to the CRCoV CD2 strain, several aa mutations were identified in the S gene of the other four strains. Specifically, CRCoV CD1, CD3, and CD4 strains share a unique aa mutation at position I/S510T; CRCoV CD3 and CD4 strains share a unique aa mutation at position V132L; and the CRCoV CD5 strain has two unique aa mutations at positions M/T74K and T972I. Notably, the majority of these aa changes (I/S510T, V132L, and M/T74K) are located in the S1 subunit.

The complete HE genes of the CRCoV CD1-CD5 strains have a full length of 1,275 bp and encode 424 aa. The nt and aa similarity between the HE genes of the five strains in this study was 100%. Compared to all complete HE coding sequences in GenBank, the nt similarity ranged from 97.3% to 99.8%, and the aa similarity ranged from 96.7% to 99.1%. Notably, three unique aa mutations (P62S, S158F, and L161F) were identified in the HE protein of the five CRCoV strains in this study. The P62S mutation is located in the esterase domain, while S158F and L161F are located in the lectin domain.

### 3.3. Selection pressure analysis

The relative dN/dS ratio of the S gene among the 42 sequences ranged from 0.179 to 0.575, which is less than 1, indicating negative selective pressure. However, site-by-site selection analysis of the four aa mutations identified in the S protein of the five CRCoV strains revealed that all mutation sites (74, 132, 510, and 972) were under positive selection. Specifically, site 510 was supported by three algorithms, sites 132 and 972 were supported by two algorithms, and site 74 was supported only by the MEM algorithms (Table 2).

**Table 2.** Selection pressure prediction analysis of positive selection pressure loci.

Gene	AA	MEME (*p* value)	FEL (*p* value)	SLAC (*p* value)	FUBAR (Post Pro)
S	74	+	–	–	–
	132	+	+	–	–
	510	+	+	–	+
	972	+	+	–	–
HE	62	+	+	–	–
	158	+	+	–	–
	161	+	+	–	–

The dN/dS ratio for the HE gene among the 39 sequences ranged from 0 to 1.214. The dN/dS ratios for the CRCoV CD1-CD5 strains, as well as the Chinese strain BJ232 (KX432213.1) and the Thai PP146 (OQ621713.1) and PP158 (OQ621714.1) strains, are all 1.214, indicating potential positive selection, this metric itself may be affected by evolutionary scale. More reliably, site-by-site selection analysis of three aa mutation sites (62, 158, and 161) identified in the HE protein of five CRCoV strains showed that all mutation sites are under positive selection. This finding is supported by two different algorithms (Table 2).

### 3.4. Structure modelling of ORF1ab and HE protein

Our analysis of CRCoV CD1-CD5 genomes unveiled distinctive mutational profiles within critical viral genes. The ORF1ab protein notably exhibited a variable number of aa substitutions across strains, yet consistently harbored a singular shared mutation (K1866R). Concurrently, the HE protein displayed a highly conserved mutational signature, characterized by three identical aa mutations (P62S, S158F, and L161F) present in all five genomes. To elucidate the structural and functional consequences of these differential and shared substitutions, molecular modeling techniques were subsequently applied.

A homology model was obtained through the Swiss-Model server using PDB ID structure 3CL4.1 from BCoV Mebus as a template. The overall structure of the CRCoV HE protein closely resembles that of the BCoV Mebus strain, assembling into homodimers composed of three modules: the membrane-proximal domain, the lectin domain, and the esterase domain. Due to the absence of suitable homologous templates, the 3D structure of ORF1ab protein was predicted using AlphaFold. Mutational analysis indicated that neither the identified substitutions in ORF1ab nor those in the HE protein induced substantial alterations in their global protein conformations. Nevertheless, DynaMut analysis revealed that multiple aa mutations in ORF1ab across the five strains (CD1-CD5) imparted localized changes in conformational stability and flexibility (Table 3). Specifically, the common K1866R mutation, observed in all five ORF1ab strains, consistently resulted in a reduction of protein stability (ΔΔG = −0.3941) and an increase in flexibility (ΔΔS _Vib_ ENCoM = 0.221).

**Table 3.** Localized changes in protein conformational stability and flexibility induced by ORF1ab amino acid mutations in CRCoV CD1-CD5 strains.

Mutant strain	Aa mutation	ΔΔG (kcal/mol)	Stability	ΔΔS _Vib_ ENCoM (kcal.mol^−1^.K^−1^)	Molecule flexibility
CD1	H1766Y	1.089	Stabilizing	−0.295	Decrease
	K1866R	−0.3941	Destabilizing	0.221	Increase
	F2848V	−0.288	Destabilizing	0.297	Increase
	A2903V	0.902	Stabilizing	−0.578	Decrease
	Y6809C	−0.033	Destabilizing	0.019	Increase
	K6810Q	−0.6	Destabilizing	0.48	Increase
	M6816V	−0.092	Destabilizing	1.084	Increase
	E6817D	0.236	Stabilizing	−0.033	Decrease
	S6820F	0.485	Stabilizing	−0.205	Decrease
CD2	P1125S	0.021	Stabilizing	0.012	Increase
	H1766Y	1.089	Stabilizing	−0.295	Decrease
	K1866R	−0.3941	Destabilizing	0.221	Increase
	D1941N	0.418	Stabilizing	−0.107	Decrease
	I6073N	−0.857	Destabilizing	0.231	Increase
CD3	K1866R	−0.3941	Destabilizing	0.221	Increase
	A2903V	0.902	Stabilizing	−0.578	Decrease
	P5538A	0.256	Stabilizing	0.057	Increase
	T5827A	0.126	Stabilizing	0.371	Increase
	D5829A	−0.662	Destabilizing	0.486	Increase
	G5833S	−0.435	Destabilizing	−0.760	Decrease
	E5835D	−0.057	Destabilizing	0.340	Increase
CD4	K1866R	−0.3941	Destabilizing	0.221	Increase
	A2903V	0.902	Stabilizing	−0.578	Decrease
	A5992N	−0.588	Destabilizing	−0.010	Decrease
CD5	H1766Y	1.089	Stabilizing	−0.295	Decrease
	K1866R	−0.3941	Destabilizing	0.221	Increase

The results from DynaMut, which predicts the effects of mutations on protein stability and flexibility, indicated that among the three aa mutations (P62S, S158F, and L161F) in the HE protein, the P62S mutation was predicted to be unstable, with a ΔΔG value of −0.152. In terms of flexibility prediction, the P62S mutation exhibited an increase in flexibility, while the S158F and L161F mutations were associated with decreased flexibility, yielding ΔΔS _Vib_ ENCoM values of 0.190, −1.572, and −0.195, respectively (Table 4).

**Table 4.** Impact of amino acid mutations on stability and flexibility of novel Chinese CRCoV HE protein.

Protein	Mutation	ΔΔG (kcal/mol)	Stability	ΔΔS _Vib_ ENCoM (kcal.mol^−1^.K^−1^)	Molecule flexibility
HE	P62S	−0.152	Destabilizing	0.190	Increase
	S158F	1.913	Stabilizing	−1.572	Decrease
	L161F	0.437	Stabilizing	−0.195	Decrease

**Figure 3.**
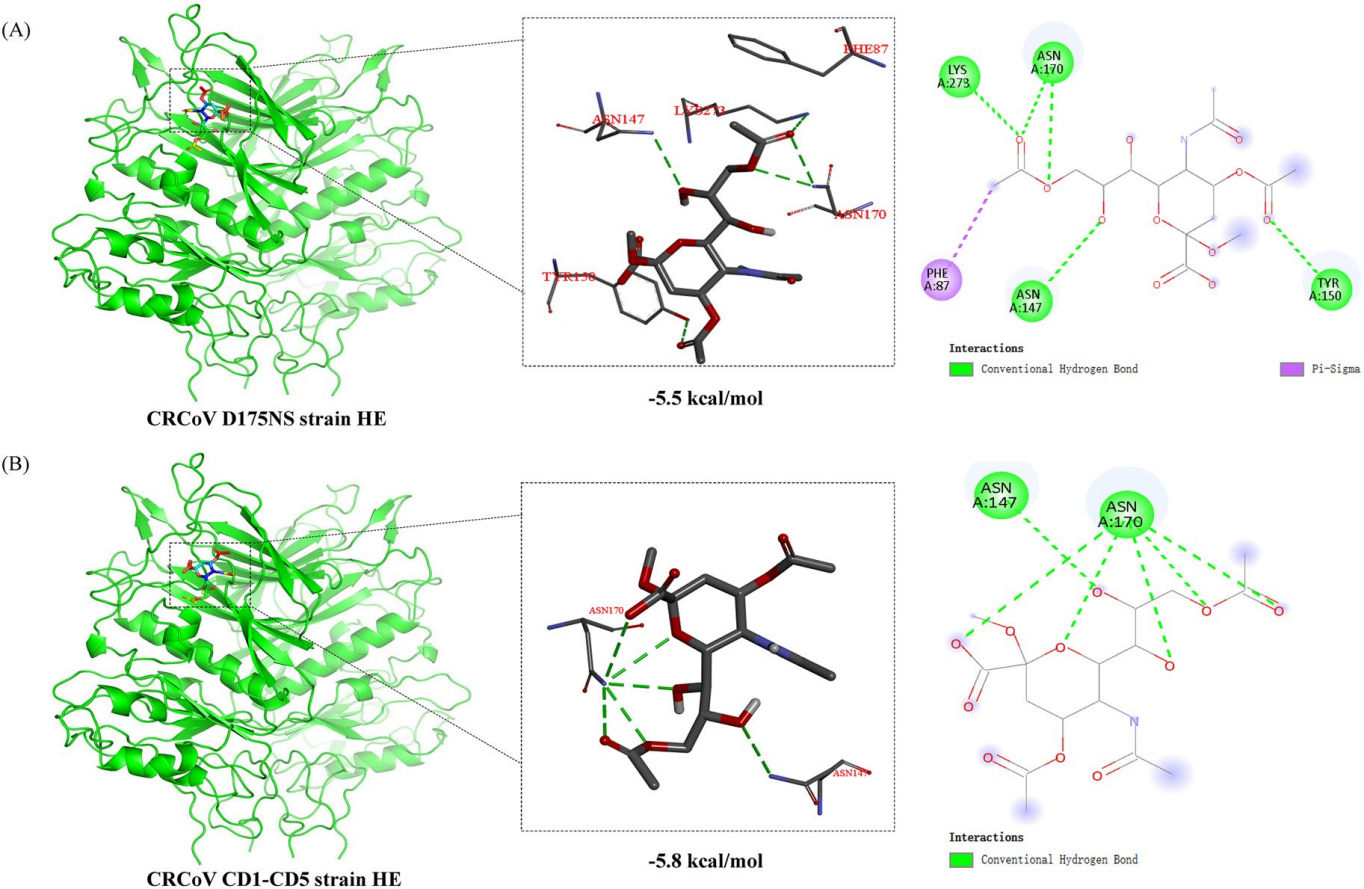
Molecular docking model of HE protein and O-acetylated silica. (A) Molecular docking structure of CRCoV D175NS strain and O-acetylated silica with a docking energy of −5.5 kcal/mol. (B) Molecular docking structure of CRCoV CD1-CD5 strains (since their amino acid sequences are the same, only one representative structure was provided) and O-acetylated salicylic acid. The docking energy is −5.8 kcal/mol. Green dashed lines represent hydrogen bonds. Violet lines represent Pi-Sigma bonds.

Molecular docking results showed that O-acetylated sialic acid can spontaneously bind to the receptor-binding domain of the CRCoV D175NS and CRCoV CD1-CD5 strains. A binding energy of less than 0 indicates that the ligand can spontaneously bind to the receptor protein, while a binding energy of less than −5 kcal/mol between the active compound and the target protein receptor is considered indicative of good binding activity. O-acetylated sialic acid binds to the strains CRCoV D175NS and CRCoV CD1-CD5 with binding energies of −5.5 kcal/mol and −5.8 kcal/mol, respectively. The binding energy values below −5 kcal/mol suggest that O-acetylated sialic acid has a strong affinity for CRCoV D175NS and CRCoV CD1-CD5 strains. Notably, the CRCoV CD1-CD5 strains exhibited a higher affinity for O-acetylated sialic acid (−5.8 kcal/mol < −5.5 kcal/mol) due to the HE mutation present in these strains (Figure 3).

### 3.5. CRCoV isolation and identification

**Figure 4.**
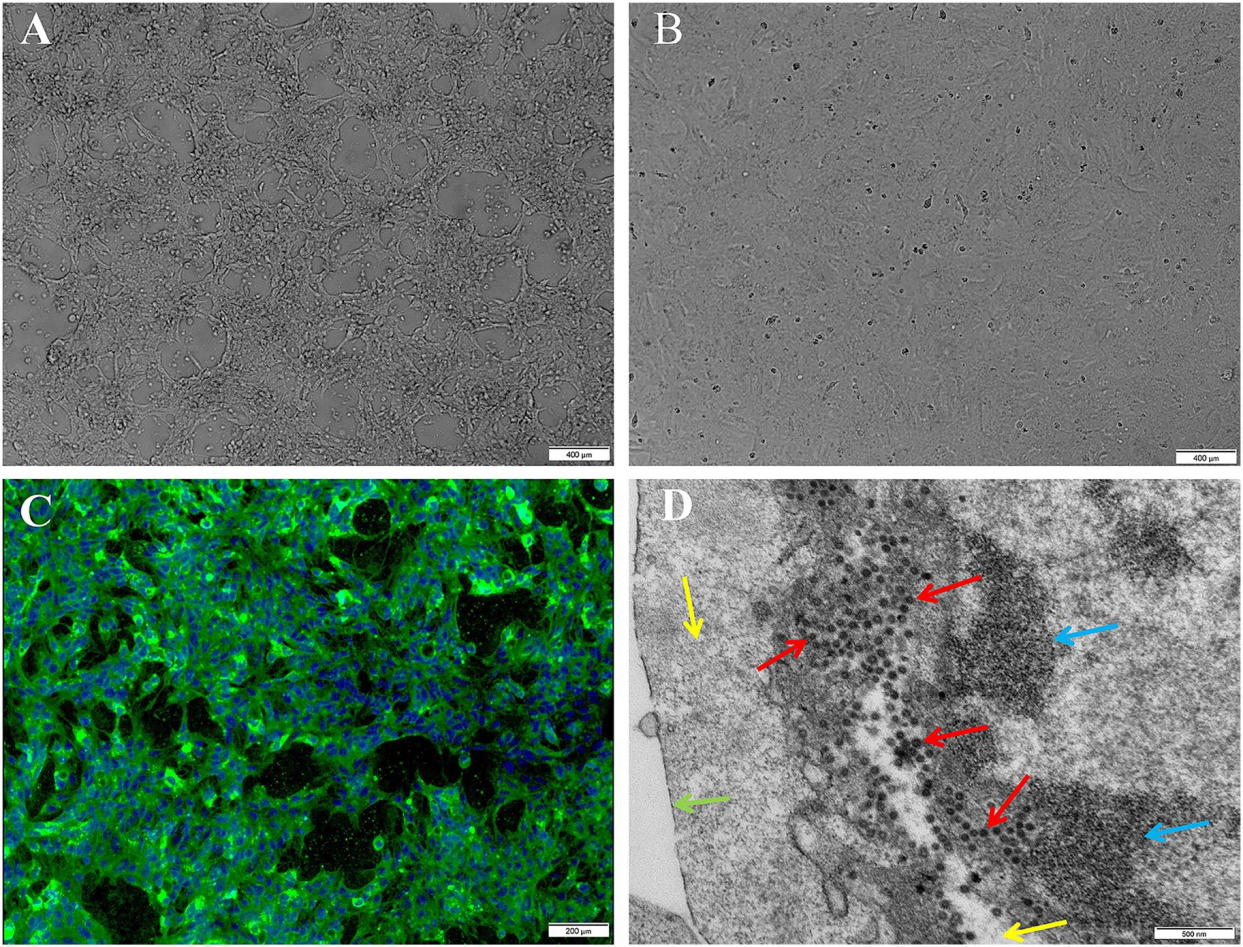
Microscopic examination of CRCoV isolated. (A) CPE observed 96 h post-inoculation, characterized by cell shrinkage and detachment. (B) Control cells after 96 h of culture, showing no CPE. (C) The IIFA of the isolate in HCT-8 cells, showing specific fluorescent signals. (D) TEM of the isolate from HCT-8 cell cultures. Red arrows indicate virus particles, blue arrows point to HCT-8 cell nuclei, green arrows indicate HCT-8 cell membranes, and yellow arrows indicate HCT-8 cell cytoplasm.

Among 15 nasal swab samples collected from pet dogs with respiratory diseases, 10 tested positive for CRCoV. These positive samples were inoculated onto HCT-8 cells and passaged blindly three times. CPE characterized by cell shrinkage and cell detachment was observed in only one sample at 96 h post-inoculation (Figure 4A); no CPE was observed in the negative control cells (Figure 4B). Stable CPE consistently appeared after 96 h during passages from the fifth to the eighth. Virus purification was performed on the sixth-generation cell culture, yielding a viral titer of 10^6^ TCID_50_/mL. No CPE was observed in the remaining nine cell culture samples. Specific fluorescent signals were detected in HCT-8-infected cells inoculated with the isolate using immunofluorescence microscopy by IIFA (Figure 4C). Ultrathin sections of the infected cells observed under TEM revealed spherical viral particles approximately 60 nm in diameter, enclosed within vesicle membranes (Figure 4D). The CRCoV isolate from was named CRCoV CD1.

### 3.6. Experimental infection of dogs

#### 3.6.1. Clinical observations

The control group remained healthy throughout the entire study duration. In contrast, dogs in the challenge group (*n*＝3) exhibited clinical symptoms as early as 4 DPI, including nasal discharge, sneezing, and coughing. These symptoms gradually worsened in the following days, accompanied by ocular discharge, peaking between 6 DPI and 9 DPI in the challenge group. By 10 DPI, the symptoms began to subside. At the end of the study (14 DPI), only very mild sneezing was observed in the challenged group, with all other symptoms resolved.

#### 3.6.2. CRCoV shedding and tissue tropism

The qRT-PCR analysis for CRCoV was conducted on both pharyngeal and nasal swabs. All dogs in the control group tested negative for CRCoV on day 1 of the study (prior to the challenge), and remained negative throughout the study period. In contrast, CRCoV shedding was detected in pharyngeal swabs and nasal swabs of the challenge group throughout the experimental period, which lasted up to 14 days.

**Figure 5.**
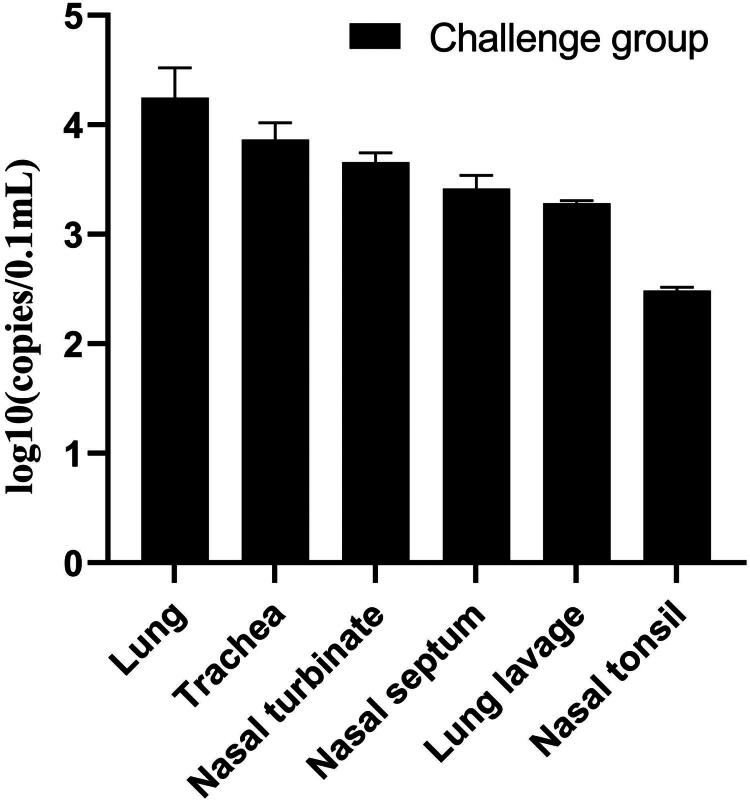
Viral genomic copy numbers in the lung, trachea, nasal turbinate, nasal septum, lung lavage, and nasal tonsil of dogs at 14 DPI.

CRCoV nucleic acids were quantified by qRT-PCR in tissues collected at autopsy on 14 DPI. In the challenge group, CRCoV nucleic acids were detected in the lungs, trachea, nasal septum, nasal turbinates, lung lavage, and nasal tonsil. The highest levels of CRCoV nucleic acids were found in the lungs, followed by the trachea, nasal turbinates, nasal septum, lung lavage, and nasal tonsil (Figure 5). Concurrently, all examined extra-respiratory tissues (heart, liver, spleen, kidneys, duodenum, jejunum, ileum, cecum, colon, and rectum) were not detected with CRCoV viral nucleic acid at the time of sampling (14 DPI). In contrast, the control group remained negative for CRCoV viral nucleic acid across all tested tissues throughout the study period.

#### 3.6.3. Histopathology

**Figure 6.**
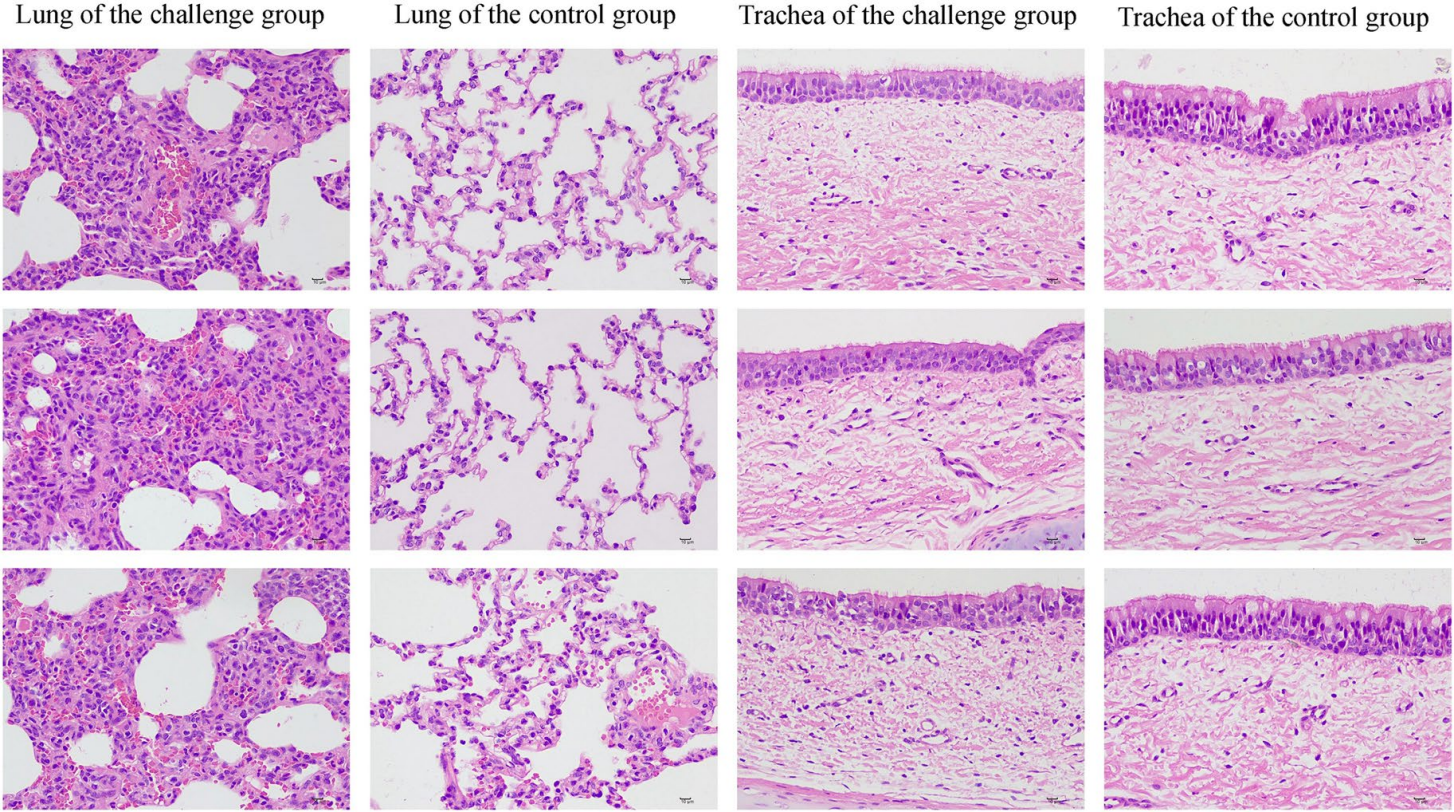
Histopathological changes in the lungs and trachea of experimental dogs.

No obvious lesions were observed in the organs with the naked eye. Pathological examinations revealed that tissue lesions were primarily concentrated in the lungs and trachea of the experimental dogs and no lesions were found in other tissues. Histopathological changes included thickening of the alveolar septa and infiltration of lymphocytes and macrophages. In the trachea, histopathological examination revealed loss of cilia, disorganization of epithelial cells, absence of goblet cells, and infiltration of inflammatory cells in the intraepithelial region and the underlying lamina propria (Figure 6).

## 4. Discussion

CIRD is a globally prevalent syndrome caused by various viral and bacterial pathogens, including CRCoV (Day et al. 2020). However, its genome characteristics and pathogenicity remain limited. In this study, we identified two consecutive nucleotide deletions in the non-coding region of CRCoV CD1-CD5 genomes, a feature consistent with the genomic characteristics of Thailand CRCoV strains sequenced between 2021 to 2022 (OQ621707.1-OQ621727.1). Notably, this specific deletion pattern has not been previously reported in Thai strains. Additionally, while a continuous nucleotide deletion was observed in the gene encoding the 4.9 kDa nonstructural protein of Thai CRCoV (OQ621707.1-OQ621727.1), this variation was absent in the CRCoV CD1-CD5 genomes analyzed here. These features were not detected in other known strains, underscoring the dynamic evolutionary processes occurring in both the non-coding and nonstructural gene regions of CRCoV. This finding warrants attention, as genomic studies of SARS-CoV-2, which also belongs to the β-coronavirus genus, have demonstrated that nt deletions in non-coding regions frequently occur within consecutive arrangements of identical nucleotides or in tandem repeats of dinucleotides and trinucleotides. This phenomenon is common in the evolutionary processes of viruses and may lead to variations in the viral genome, allowing adaptation to different environments or hosts (Rogozin et al. 2023).

Previous studies have shown that mutations within genomic non-coding regions can alter the structure of cis-regulatory elements residing in these regions, thereby affecting multiple aspects of gene expression, such as transcriptional efficiency, mRNA stability, and translation (Sabarinathan et al. 2013). On this basis, our findings concerning CRCoV non-coding region deletions offer significant insights into their potential regulatory roles. Specifically, the observed reduction in core promoter elements and the concomitant decrease in binding stability (as evidenced by an increase in average minimum free energy) indicate that deletions within CRCoV non-coding regions may further potentially affect gene transcription, mRNA translation and next protein expression, thereby influencing CRCoV’s biological characteristics. While these initial findings warrant further functional validation, future studies should experimentally validate these predicted regulatory effects and comprehensively elucidate their contribution to CRCoV molecular biology and evolution, thereby advancing our understanding of coronavirus adaptation and pathogenesis.

Furthermore, previous studies have demonstrated that deletions, mutations, and recombinations in the CRCoV genome play an important role in viral evolution (Erles et al. 2003, 2007; Lorusso et al. 2009; Poonsin et al. 2023). Further illuminating these viral evolution dynamics, complete-genome phylogenetic analysis revealed that the CRCoV CD1-CD5 strains formed a unique small branch, exhibiting significant genetic distance from other Chinese strains. Intriguingly, CRCoV CD1-CD5 Chinese strains showed a distinct clustering with previously reported Thai CRCoV strains (OQ621715.1-OQ621727.1). Despite the significant geographical distance, this close genetic relationship prompts a deeper consideration of the potential epidemiological and evolutionary mechanisms facilitating such long-distance viral dissemination. One primary potential explanation for this phylogenetic association is the movement of canine hosts between these two regions. The globalized nature of modern society involves substantial international trade and movement of companion animals, providing plausible routes for an infected, potentially asymptomatic, dog to introduce the virus into new susceptible populations, amplified by high-density environments like kennels or international dog shows.

Beyond simple linear transmission, the evolutionary dynamics of CRCoV are also profoundly shaped by recombination events, a known mechanism for generating novel variants (Erles et al. 2007; Lu et al. 2016). Indeed, both inter- and intra-species recombination have been documented in CRCoV, with examples including the recombinant CRCoV K37 strain from South Korea derived from parental strains CRCoV BJ232 and Bovine Coronavirus (BCoV) Quebec (Lu et al. 2016), and Thai PP 158_THA_2015 strain, which acted as a potential parental strain for recombinant CRCoV USA 3 (Erles et al. 2007). Therefore, the clustering of ORF1ab, S, and HE genes of our Chinese CD1-CD5 strains with the Thai strains could plausibly reflect an undetected recombination event within our dataset. While we did not identify such events, the scarcity of complete CRCoV genomes in GenBank significantly limits recombination analysis; detections can fail if parental strains share highly similar patterns or are unavailable. This mechanism is a key driver of CRCoV diversity and a plausible explanation for complex phylogenetic patterns globally. These results provide insights into CRCoV evolution, emphasizing that genetic distance and diversity are driven by multiple factors, including mutations, selective pressures, and genetic recombination (; Forni et al. 2017; Goldstein et al. 2022; Li et al. 2024;  Shah et al. 2025). This underscores the concept of the global canine population as a single, interconnected reservoir for pathogens, highlighting the limitations of regional surveillance and the urgent need for a globally coordinated genomic surveillance strategy for companion animal viruses.

The ORF1ab gene, which constitutes about 75% of the coronavirus genome, encodes a series of non-structural proteins that assemble to facilitate viral replication and transcription (Li et al. 2020). Prior investigations into related coronaviruses, such as HCoV-OC43, SARS-CoV and MHV, have established that mutations within ORF1ab can modulate viral biological characteristics, including adaptability and pathogenicity (Sperry et al. 2005; Menachery et al. 2014; Forni et al. 2016; Kindler et al. 2017). However, the functional implications of the distinct mutations observed in CRCoV CD1-CD5 strains (Table 1) are presently undefined. Analysis of these mutations, informed by molecular conformational studies, suggests that aa substitutions within ORF1ab in these CRCoV strains may elicit localized alterations in conformational stability and flexibility. Such structural modifications possess the potential to influence protein biological function. Consequently, these identified mutations warrant consideration as prospective targets for future inquiry into their contributions to viral evolution or inherent biological properties. Therefore, the deployment of reverse genetics or replicon systems is imperative for a comprehensive elucidation of their potential impact on viral evolution and biological characteristics.

The coronavirus S protein is critical for mediating coronavirus entry (Li 2016). In this study, four unique aa mutations (T972I, I/S510T, V132L, and M/T74K) were identified in the S protein of CRCoV CD1-CD5. These mutations were predicted to be under positive selection pressure, with I/S510T, V132L, and M/T74K located in the S1 subunit, and T972I located in the S2 subunit. The S1 subunit plays a role in receptor recognition and induces the production of neutralizing antibodies (Zhu et al. 2022); therefore, changes in the S1 subunit can affect viral antigenicity and pathogenicity. Concurrently, the S2 subunit functions as a mediator of fusion between the virus and the cell membrane, with both the S1 and S2 subunits being critical in determining host range and tissue tropism (Li et al. 2024). Studies of MHV indicate that mutations in the S2 subunit can disrupt S1 subunit-receptor binding, thereby affecting the virus’s ability to expand its host range (de Haan et al. 2006; McRoy and Baric 2008). Thus, an in-depth investigation of these mutations functional significance will provide valuable insights for the development of vaccines and therapeutic strategies.

The HE protein, present in species of the β-coronavirus genus, is a type 1 transmembrane protein responsible for recognizing cell receptors that coronaviruses use to infect susceptible cells. It also interacts with various forms of sialic acid (Lang et al. 2020;  Zhao et al. 2025). Moreover, due to the acetylesterase activity of the HE protein, it can remove acetyl groups from O-acetylated sialic acids, suggesting a potential role as a receptor-destroying enzyme (Vlasak et al. 1988; Huang et al. 2015). In this study, the HE proteins in CRCoV CD1-CD5 were found to be under positive selection pressure, specifically showing that the CRCoV CD1-CD5, Chinese BJ232 strain(KX432213.1), Thai PP146 strains (OQ621713.1), and PP158 (OQ621714.1) all exhibited a dN/dS ratio of 1.214, suggesting positive selection during their evolutionary processes. Additionally, a site-by-site selection analysis of the three aa mutation sites (62, 158, and 161) identified in the HE protein revealed that all mutation sites are under positive selection. Although the exact role and function of the CRCoV HE protein have not been fully elucidated, related studies on the HE proteins of BCoV and HCoV-OC43, which are genetically similar to CRCoV, have been conducted (Erles et al. 2007). Research on the BCoV HE protein has revealed that its structural domains primarily include the membrane-proximal domain, esterase domain, transmembrane domain, and lectin domain. There are six loops exposed on the surface of the HE protein, including the E1-loop located in the esterase domain, and the R1-loop, R2-loop, R3-loop, R4-loop, and RBS‐hairpin located in the lectin domain (Zeng et al. 2008). In this study, three unique aa mutations (P62S, S158F, and L161F) were identified in the HE protein of the novel CRCoV strain (CRCoV CD1-CD5). The P62S mutation is located in the esterase domain, while S158F and L161F are located in the lectin domain, with L161F specifically situated in the R1-loop.

The esterase domain in coronavirus HE proteins exhibits receptor-destroying enzyme activity, facilitating the cleavage of sialic acid receptors. This activity aids in the release of the virus from the host cell surface, promoting viral spread (Schultze et al. 1991). The novel mutation in the Chinese CRCoV HE protein introduces serine at position 62, replacing proline. Proline’s cyclic structure confers conformational rigidity; thus, this mutation was predicted to decrease local conformational stability and increase the protein’s flexibility. This increased flexibility may enable more dynamic conformational changes during receptor binding, potentially modulating its interaction with the receptor. Similarly, the lectin domain in coronavirus HE proteins possesses receptor-binding activity, enabling the recognition of sialic acid receptors. This recognition not only facilitates viral release from the host cell surface but also plays a critical role in viral attachment, entry, replication, and dissemination (Bakkers et al. 2017). The S158F and L161F mutations may reduce the protein’s local flexibility and stability, which could alter the HE protein’s ability to bind to the receptor. Previous studies on BCoV and MHV HE proteins indicates that the L161I mutation in MHV diminishes the receptor-binding ability of HN protein (Langereis et al. 2012). Molecular docking analysis suggests that, in the Chinese strain CRCoV CD1-CD5, the S158F and L161I mutations in the HE lectin domain are associated with improved docking scores, indicating a potential increase in affinity for O-acetylated sialic acid. However, this finding is based exclusively on molecular docking, which lacks direct biochemical validation and fails to capture full protein dynamics, glycosylation, or the complexity of the host cellular environment; therefore, further experimental validation is necessary to substantiate its biological significance. Notably, HCoV-OC43, thought to have originated from cross-species transmission of BCoV, displays unique aa mutations at positions 158 (S158A) and 161 (L161F) during its evolution (Bakkers et al. 2017). These cumulative HE protein mutations are increasingly recognized as part of coronavirus adaptive evolution of coronaviruses (Bakkers et al. 2017). Therefore, further investigation into the impact of these unique aa mutations in the HE protein on viral pathogenicity and adaptability is essential for understanding their effects on viral biological properties and interactions with host cells.

Considering the possible effects of the genomic variants described above, it is particularly important to conduct an in-depth study of the isolation of CRCoV. Previous studies have shown that isolation and culture of CRCoV are challenging (Erles et al. 2007; Lu et al. 2016). In this study, one strain, CRCoV CD1, was successfully isolated using HCT-8 cells, whereas attempts to isolate other samples were unsuccessful. We analyzed the potential reasons for these isolation failures. Foremost, cell tropism plays a crucial role. Despite the employment of various cell lines (A72, BHK-21, CHO, DH82, MDBK, MDCK, Vero, and HCT-8) in reported CRCoV isolation attempts, HCT-8 is frequently the sole cell line cited for successful isolation (Erles et al. 2007; Lu et al. 2016). This observation suggests that the HCT-8 cell line might not be optimally permissive for all CRCoV strains, or that certain strains possess a very narrow host cell tropism that our current *in vitro* system cannot fully support. Furthermore, an insufficient initial viral load in the collected samples, compounded by potential compromises to viral viability during transport and processing, may have collectively hindered successful *in vitro* cell infection and subsequent propagation. While genomic mutations affecting viral replication or receptor binding (as previously discussed concerning ORF1ab, M, N, S, and HE proteins) could theoretically contribute to the recalcitrance of certain strains to isolation, the inherent biological attributes of CRCoV, particularly its cell tropism, are widely acknowledged as the principal impediments to achieving robust *in vitro* culture.

Pathogenicity experiments showed that the isolate was capable of inducing respiratory tract infections in experimental dogs, exhibiting typical symptoms of upper respiratory tract infections, including increased nasal and eye secretions, sneezing, and coughing. This confirms its ability to cause respiratory tract diseases in dogs and aligns with clinical signs reported in prior studies of CRCoV infection (Mitchell et al. 2013). The observed data support a primary respiratory tropism for CRCoV. Nevertheless, given the lack of earlier time-point sampling, transient viremia or an initial, brief dissemination to extra-respiratory tissues followed by rapid clearance cannot be entirely ruled out. Combined with the results of PCR testing and histopathological examination revealed that the isolate primarily proliferated in the lungs and trachea, which is different from previous studies indicating that CRCoV strains predominantly proliferate in the trachea and nasal cavity (Mitchell et al. 2013). This preliminary observation, coupled with molecular docking results, leads us to hypothesize that the isolate from this study may exhibit an altered tissue tropism, potentially linked to mutations in its S or HE proteins. We further hypothesize that these mutations may have influenced the virus’s binding preferences for various tissue cell receptors. However, the limitations of this preliminary pathogenicity study, including a relatively small sample size and short observation period, mean that definitive claims, particularly regarding a tissue tropism shift, are premature. Tissue viral load variability can be influenced by factors like sampling timing and infection dose. While our findings suggest a potential alteration, comprehensive validation requires further investigation. Therefore, future studies are warranted to rigorously investigate the effects of these mutations on viral biology and to confirm tissue tropism. Specifically, longitudinal time-course experiments and *in situ* hybridization analyses will be crucial to track viral distribution and replication kinetics within different tissues, providing more definitive evidence for or against a tissue tropism shift. Furthermore, to comprehensively characterize disease progression and allow for better extrapolation of results to natural infection processes, future investigations will expand sample cohorts and incorporate longitudinal monitoring.

## 5. Conclusion

This study characterized five complete CRCoV genomes from China (PQ725948-PQ725952), revealing two consecutive nucleotide deletions in non-coding regions, which suggest dynamic evolutionary processes in CRCoV. Phylogenetic analyses of the ORF1ab, HE, and S genes demonstrated high consistency with the complete genome tree, underscoring the pivotal role of these genes in CRCoV evolution. Furthermore, unique aa mutations were identified in the ORF1ab, HE, S, M, and N proteins. Molecular docking results indicate a potential increase in the binding affinity of HE for the receptor due to the S158F and L161F mutations. Notably, a pathogenic CRCoV strain was successfully isolated, and experimental infection studies demonstrated its ability to induce pneumonia and tracheal cilia loss in canines. These findings collectively highlight the emergence of a novel CRCoV variant in China, characterized by distinct genetic and pathogenic features. Ongoing surveillance is warranted to assess the epidemiological impact of this variant.

## Supplementary Material

Supplementary_Figure_1.tif

Supplementary_Table_2.xls

Supplementary_Table_3.xls

Supplementary_Table_1.xls

## Data Availability

The authors confirm that the data supporting the findings of this study are available within the article and its supplementary materials.
