# Supplementary figures and images for: Characteristics of an emerging canine respiratory coronavirus in China
Source: Vet Q. 2025 Oct 17;45(1):2574506. doi: 10.1080/01652176.2025.2574506 (PMC12536626; doi:10.1080/01652176.2025.2574506)

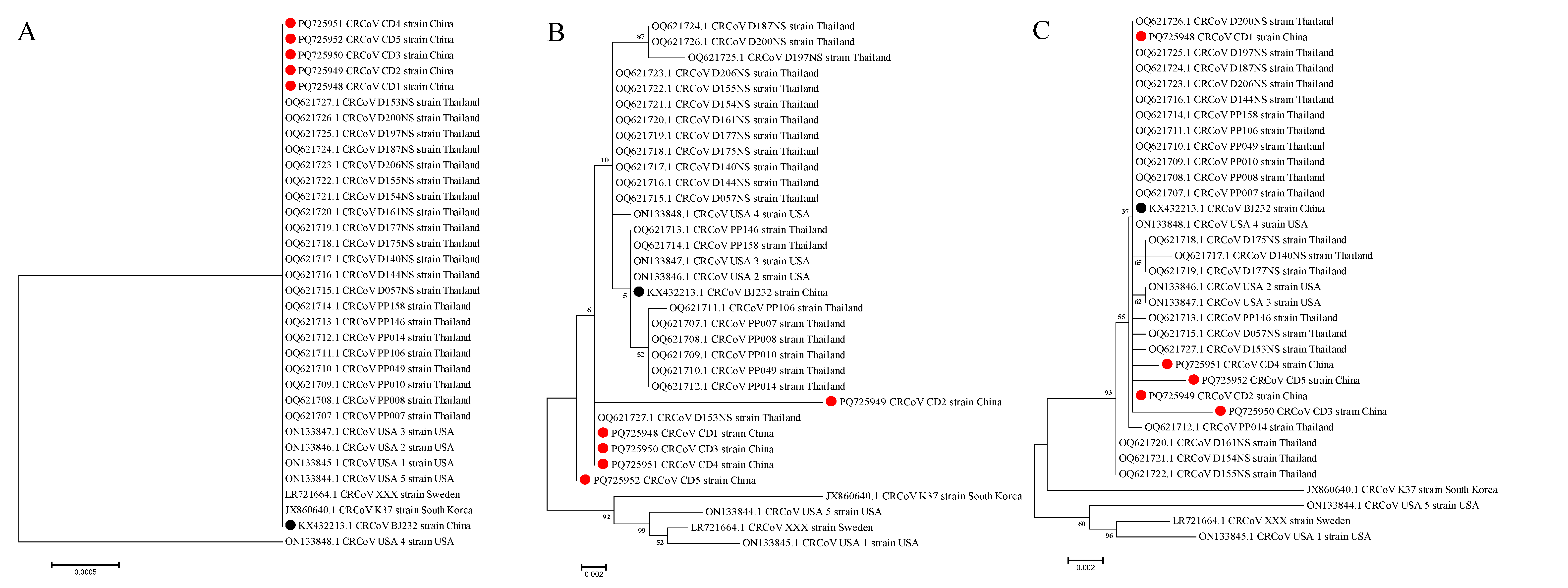

Supplement: Supplementary_Figure_1.tif [file TVEQ_A_2574506_SM2758.tif]
